# Bisphenol A exposure during early pregnancy impairs uterine spiral artery remodeling and provokes intrauterine growth restriction in mice

**DOI:** 10.1038/s41598-018-27575-y

**Published:** 2018-06-15

**Authors:** Judith Elisabeth Müller, Nicole Meyer, Clarisa Guillermina Santamaria, Anne Schumacher, Enrique Hugo Luque, Maria Laura Zenclussen, Horacio Adolfo Rodriguez, Ana Claudia Zenclussen

**Affiliations:** 1 Experimental Obstetrics and Gynecology, Medical Faculty, Otto-von-Guericke University, Magdeburg, Germany; 2 Universidad Nacional del Litoral, Facultad de Bioquímica y Cs. Biológicas, Instituto de Salud y Am biente del Litoral, UNL-CONICET, Santa Fe, Argentina

## Abstract

Endocrine disrupting chemicals are long suspected to impair reproductive health. Bisphenol A (BPA) has estrogenic activity and therefore the capacity of interfering with endocrine pathways. No studies dissected its short-term effects on pregnancy and possible underlying mechanisms. Here, we studied how BPA exposure around implantation affects pregnancy, particularly concentrating on placentation and uterine remodeling. We exposed pregnant female mice to 50 µg/kg BPA/day or 0.1% ethanol by oral gavage from day 1 to 7 of gestation. High frequency ultrasound was employed to document the presence and size of implantations, placentas and fetuses throughout pregnancy. Blood velocity in the *arteria uterina* was analyzed by Doppler measurements. The progeny of mothers exposed to BPA was growth-restricted compared to the controls; this was evident *in vivo* as early as at day 12 as analyzed by ultrasound and confirmed by diminished fetal and placenta weights observed after sacrificing the animals at day 14 of gestation. The remodeling of uterine spiral arteries (SAs) was considerably impaired. We show that short-term exposure to a so-called “safe” BPA dose around implantation has severe consequences. The intrauterine growth restriction observed in more than half of the fetuses from BPA-treated mothers may owe to the direct negative effect of BPA on the remodeling of uterine SAs that limits the blood supply to the fetus. Our work reveals unsuspected short-term effects of BPA on pregnancy and urges to more studies dissecting the mechanisms behind the negative actions of BPA during early pregnancy.

## Introduction

Living organisms are daily exposed to endocrine disrupting chemicals (EDCs) that are exogenous, mostly man-made substances that exhibit hormonal activity. They are therefore capable of interfering, mostly in a negative way, with normal endocrine pathways and are thus called environmental estrogens or xenoestrogens. EDCs in particular interfere with processes regulated by endogenous estrogens; EDCs can adversely affect estrogen-mediated reproductive processes^1^.

Bisphenol A (BPA) is a chemical compound used worldwide in the industrial production of polycarbonate plastics and epoxide resins. Polycarbonates are employed for the production of objects of daily use like for example food containers, microwave dishes, children’s toys and plastic bottles. Epoxide resins are mainly used to coat food and beverage cans. 1.15 million tons of BPA are produced in Europe every year (Federal Environment Agency 17^th^ July 2014) and 5.5 million tons of BPA were produced in 2011 worldwide^2^. BPA is one of the most prominent and best studied EDC^3–5^. Independently of the source, free BPA was shown to leach from these polymers under normal conditions^6–8^. Moreover, heating or boiling food or beverage containers increases BPA release from polycarbonate plastics and epoxy resins^9,10^. In humans, the major uptake source of BPA is food and beverages (WHO 2012). BPA has been found in serum of persons of all ages. In samples from pregnant women different concentrations of BPA were reported^11–13^. As BPA can cross the placenta, its presence has been demonstrated in umbilical cord blood, amniotic fluid and fetal plasma^13–15^. The concentration of BPA in amniotic fluid is even higher than in plasma from pregnant women, reaching levels of 8.3 ng/mL at 15–18 weeks of^12^ or 7.75 ng/mL at full term pregnancies^16^. It has been calculated that a developing human fetus may be exposed to free BPA in the 0.2–9.2 ng/mL range^13,17^. These reports constitute an alarming fact if we take into account that BPA exhibits a u-shaped dose-response curve and in consequence could exhibit significant effects already at low concentrations. Thus, intrauterine life represents a critical period of exposure not only because BPA can reach the fetus but, most importantly, because of the hypersensitivity of developing tissues to low levels of estrogens^18,19^. Understanding the effects of EDCs during pregnancy and their short-, mid- and long-term consequences for health is of fundamental importance. However, despite the consensus regarding the wide exposure to BPA and its negative consequences in multiple systems, the long-term consequences of exposure to EDCs during fetal life are controversially discussed and not studied in depth. In particular, no detailed studies are available concerning BPA exposure around the implantation window and how this affects pregnancy.

As a matter of fact, BPA may have direct effects on pregnancy outcome as it is known to affect development, metabolism and reproductive processes^2^. Epidemiologic studies show an association between BPA and adverse perinatal, childhood, and adult health effects. BPA exposure was associated with hepatic cancer^20^, diabetes and obesity^21^ as well as with cardiovascular diseases^22^. In addition, prenatal exposure to BPA was related with increased risk of childhood asthma^23^. Urinary BPA levels were suggested to negatively correlate with semen quality^24^. BPA was also suggested to have adverse effects on male sexual function^25^. In females, serum BPA levels were found to correlate with the occurrence of miscarriages^26^ or even infertility^27^. In a Dutch cohort study, exposure to BPA was associated with lower fetal growth rates and lower weight at birth^28^. However, other studies report no association between exposure to BPA and birth weight^29^. A clear message obtained from bigger cohorts is urgently needed. Evidence regarding effects of pre- or perinatal BPA exposure is also contradictory in animal models. Although many studies have reported that low-dose BPA application did not affect the number of live pups^30–34^ in mice and rats, other reports suggest that low-dose BPA exposure may be associated with decreased number of live pups in mice^35^ and rats^36^. Overall, the scientific findings concerning the health risks due to BPA exposure during pregnancy are rather descriptive and controversial both in epidemiologic studies and animal models. Subsequent investigations of the effects of BPA on reproductive processes as well as consequences for fetuses and the underlying mechanisms are urgently needed. Here, we aimed to contribute to this knowledge gap by investigating the impact of BPA exposure during early pregnancy. Pregnant C57BL/6 mice were given 50 µg/kg BPA/day from gestation day (gd) 1 to gd7. BPA was administrated by oral gavage to mimic possible risks of diet-related BPA exposition. We chose 50 µg/kg BPA/day as the dose to apply as this concentration is considered as appropriate for the daily intake according to the Environmental Protection Agency^37^. We followed up both fetal and placental development *in vivo* by serial high frequency ultrasound measurements. Subsequently, we sacrificed the animals at day 14 of pregnancy, determined implantation and abortion rates and recorded the weights of fetus and their placentas. BPA exposure at early pregnancy stages provoked intrauterine growth restriction (IUGR) that went in hand with suboptimal remodeling of the uterine SAs.

## Materials and Methods

### Animals

Female C57BL/6 and male BALB/c mice were purchased from Janvier Labs (France) and arrived to our animal facility at the age of 8 to 11 weeks. Females were kept in groups of 2–4 animals per cage. Mice received water and food *ad libitum* and were housed with a 12 h light/dark cycle (7 am–7 pm) at 22 ± 2 °C and an air humidity of 40–60%. Experiments started after 2 weeks of acclimatization.

### Experimental design and sample collection

Animal experiments were performed according to the institutional guidelines upon ministerial approval (Landesverwaltungsamt Sachsen Anhalt: 42502-2-1296UniMD, Magdeburg, Germany). The experiments were conducted by authorized persons according to the Guide for Care and Use of Animals in Agriculture Research and Teaching. Female C57BL/6 mice were mated to BALB/c males in order to have an allogeneic mating that mirrors the mammalian situation in nature, including human beings. The day of plug was defined as gestation day (gd) 0. From gd1 females were given daily 50 µg/kg BPA/day diluted in 0.1% ethanol for 7 days by oral gavage. Control mice received 0.1% ethanol using the same procedure. Implantation number and size was followed up throughout pregnancy by high frequency ultrasound. Pregnant females were sacrificed on gd5, gd10 or gd14. The number of implantations was recorded for animals sacrificed on gd5. For those animals sacrificed on gd10 and gd14, implantation and abortions as well as the weight of fetuses and placentas were recorded. Pregnant uteri were dissected and a single implantation from each female at gd10 was dissected and fixed in a 4% paraformaldehyde (PFA) solution containing sucrose.

### High frequency ultrasound examination

In order to analyze intrauterine embryonic and fetal development, ultrasound examinations were performed at gd5, 8, 10, 12 and 14 employing a Vevo 2100 System (Fiji Fil Visualsonics Inc). Mice were anesthetized using isoflurane in an airtight box. Having entered loss of consciousness, mice were fixed in dorsal position. We employed a depilatory cream for ventral fur removal. Depending on the gd, implantation size (gd5, 8, 10, 12), placental area, -thickness, and -diameter (gd10, 14) or blood flow in the in the *arteria uterina* (uterine artery) (UA, gd5, 8, 10, 12, 14) were recorded. Peak systolic velocity (PSV) and end diastolic velocity (EDV) were defined. Besides, the resistance index (RI) and the pulsatility index (PI) were measured. Analysis of the recorded parameters was done with the help of the Vevo 2100 software.

### Measurement of fetal and placental weight

For documenting placental and fetal weight at gd14, the bicornial uterus of each mouse was collected and opened lengthwise. Fetuses and placentas were separated from surrounding decidual tissue and yolk sac. Next, the weight of each fetus and each corresponding placenta could be determined by using a scale (Kern & Sohn GmbH, minimum: 0.02 g, sensitivity: 0.001 g). Fetal growth restriction was defined as the weight below the 10^th^ percentile when compared to weights recorded for animals of the control group^38^.

### Histology

Implantations were fixed in 4% PFA containing 0.1 M saccharose (pH 7.4) for 6 h by gently shaking at room temperature (RT). Before overnight storage at 4 °C, they were transferred to 70% ethanol. The next day, the implantation specimen was dehydrated by ascending ethanol series and incubated in xylene before paraffin embedding. 5 µm slides were cut for further Haematoxylin and Eosin staining (H/E; for spiral artery analysis), immunofluorescence staining for smooth muscle actin (SMA; for spiral artery analysis) or Dolichus Biflorus Agglutinin (DBA) lectin staining for the detection of uterine Natural killer cell (uNK) analysis). To analyze the presence of uMCs, toluidine blue staining was performed as follows: pregnant uteri at gd5 were incubated in 96% ethanol. For dehydration, samples were kept in 100% ethanol for two hours, before being transferred into xylene for another 2 h and subsequent paraffin embedding. H/E, IF, DBA lectin and toluidine blue staining were done as described before^39^.

### Analysis of uterine spiral arteries (uSAs)

Parameters defining uSA remodeling were analyzed in H/E stained slides of paraffin-embedded implantations obtained at gd10. For more details as how samples were obtained for localizing SAs please see^40^. By using the Zeiss light microscope, 3–8 uSAs were identified and analyzed in the *decidua basalis* of one implantation per mouse. By using the menu function “Kontur (spline)” of the AxioVision Rel. 4.8 program, the outer and the lumen circumferences of 3–8 arteries per animal were measured in 20× magnification. The obtained values were used for calculation of the diameter (diameter = circumference/π), the wall-to-lumen ratio (uSA diameter/lumen diameter) as well the wall thickness (uSA diameter - lumen-diameter)/2). Mean values of the parameters mentioned above were calculated for each animal.

### Quantification of uMCs and uNKs

uMCs were quantified at gd5 in toluidine blue-stained uterine sections whereas uNKs were quantified in DBA-lectin-stained implantation samples by employing a micrometer eyepiece which was inserted into a light microscope. The micrometer eyepiece was positioned in ten different spots within the stained sample. The 10 × 10 single squares represents 60 × 60 µm in a 20× magnification, thus an area of 0.36 mm² could be examined. After calculating the average of 10 single squares, and considering the area measured, the amount of uMCs/uNKs per 1 mm² was determined. DBA lectin and toluidine blue staining were carried out as described before^39^.

### Statistics

Distribution of the data sets (normal or not normal) was assessed with the D’Agostino Pearson-Omnibus test before analyzing the data with either parametric or non-parametric tests. Depending on their distribution data are presented as means ± S.E.M or medians. Number of mice, samples, or experiments performed as well as the used statistical test and the according *P* values are indicated in the corresponding figure legends. GraphPad Prism 5.0 was used to perform the statistical analyses. Before analyzing the data we consulted the Statistical Department of our Faculty (Institut für Biometrie und Informatik, Medizinische Fakultät, OVGU, Magdeburg) about the appropriate test to employ for each experiment.

## Results

### BPA-treatment did not influence implantation numbers or sizes at gd5

**Figure 1:**
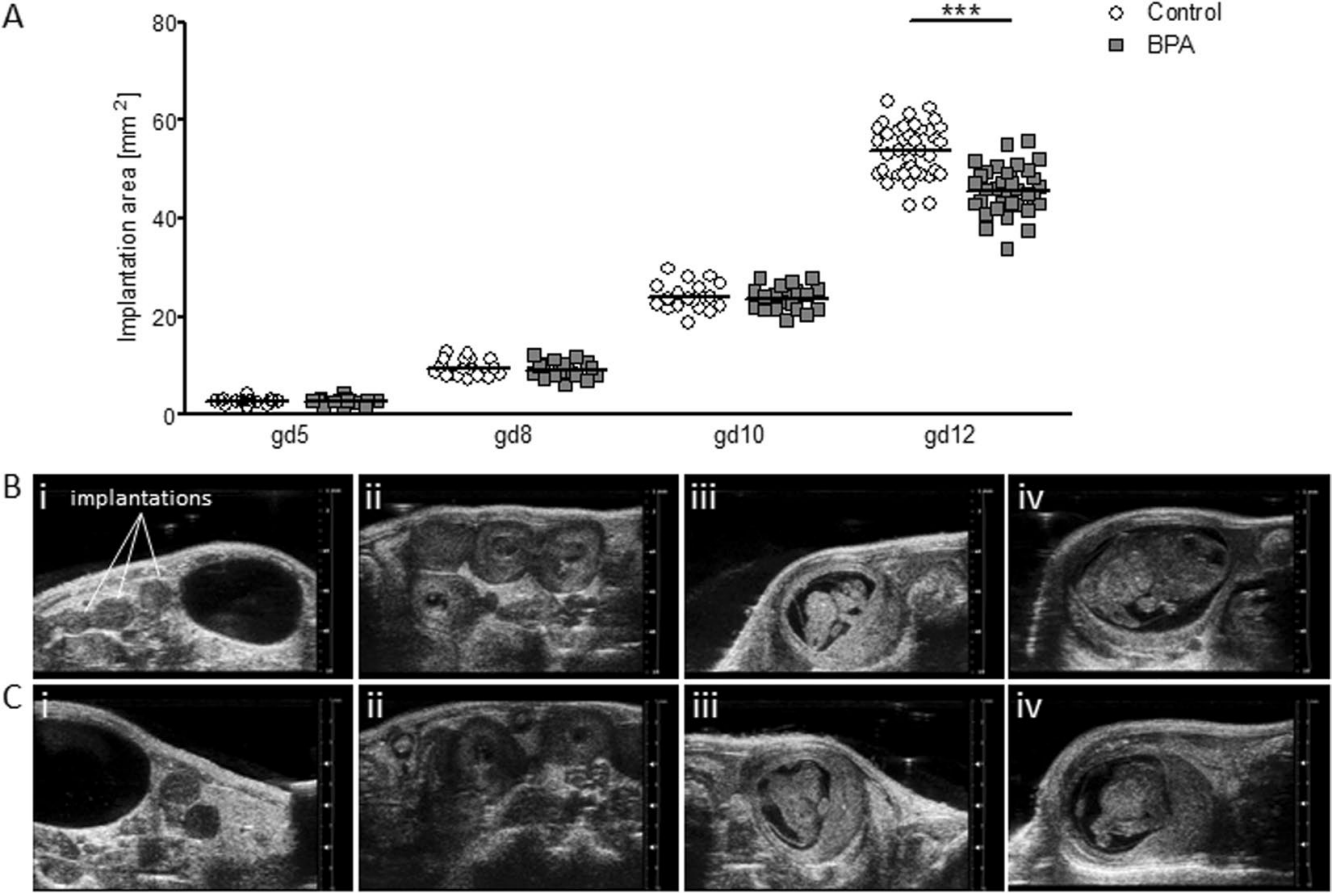
Decreased implantation size in BPA-treated mice at gd12. (**A**) Implantation areas of single implantations of control (mice: n = 5–6; implantations: n = 16–39) and BPA-treated C57BL/6J (mice: n = 5–6; implantations: n = 17–35) mice at gd5, 8, 10, and 12. Implantation sites are presented as individual values and means. Statistical differences were determined using unpaired *t*-test (***P < 0.001). Representative 2D greyscale ultrasound image from control (**B**) and BPA-treated C57BL/6J mice (**C**) at gd5 (i), gd8 (ii), gd10 (iii), and gd12 (iv). gd, gestation day.

**Figure 2:**
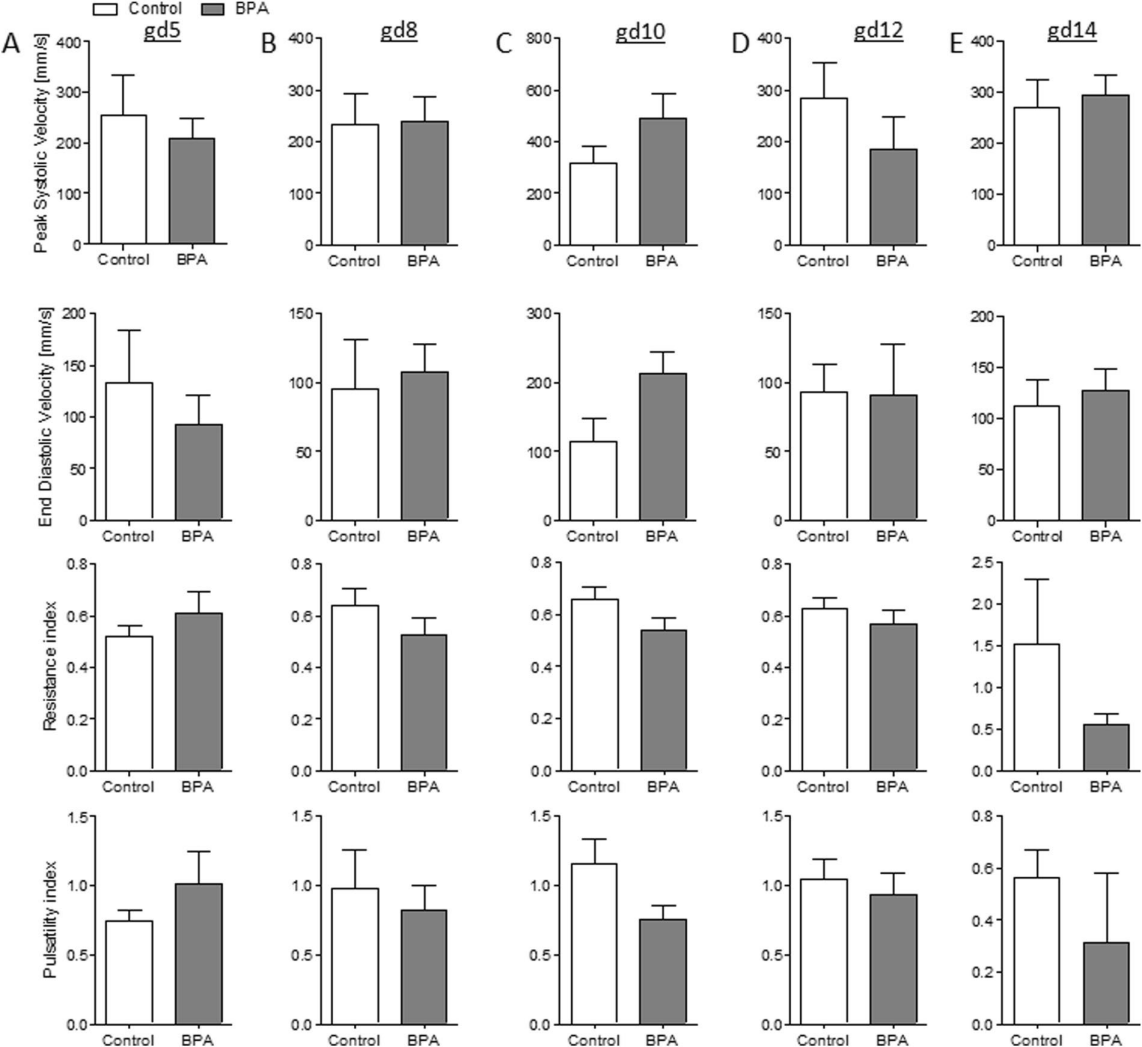
Comparable maternal uterine artery (UA) velocities in control and BPA-treated mice. Peak systolic velocity, end diastolic velocity, resistance index, and pulsatility index of UAs from C57BL/6J (n = 5) and BPA-treated C57BL/6J (n = 5) mice at gd5 (**A**), gd8 (**B**), gd10 (**C**), gd12 (**D**), and g14 (**E**). Data are presented as means with S.E.M. Statistical analysis was performed using the Mann-Whitney-*U* test. UA, uterine artery; gd, gestation day.

To understand whether the implantation process itself is affected by exposure to BPA, we determined the number of implantations and their sizes at gd5 in animals that were treated with BPA from gd1 on. The BPA dose employed for oral gavage was 50 µg/kg BPA/day; considered as the maximally allowed safe dose. Implantation size (Fig. 1A,B) were comparable between BPA-treated animals and their controls as analyzed by high frequency ultrasound and so were the blood velocity values of the UA (PSV, EDV, RI, PI) (Fig. 2A). Our results suggested no negative effect of BPA on implantation, which goes in hand with comparable implantation numbers after sacrificing the animals at gd5 (Fig. S1).

### Fetuses from BPA-treated mothers manifested IUGR as early as at gd12 as documented by ultrasound

**Figure 3:**
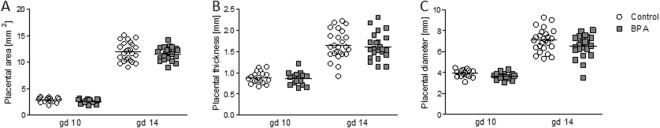
Comparable placental parameters in control and BPA-treated mice at gd10 and gd14. Placental area (**A**), placental thickness (**B**), and placental diameter (**C**) from the progenies of C57BL/6J (*n* = 5) or BPA-treated C57BL/6J (*n* = 5) mice at gd10 and gd14. Placental parameters are presented as individual values and means. Statistical differences were determined using unpaired *t*-test. gd, gestation day.

Fetal and placenta growth were followed up by serial ultrasound measurements. Blood velocity parameters of the maternal UA were evaluated by Doppler measurements. At gd8, implantation areas were comparable between the BPA-treated and control animals (Fig. 1A,B) and so were UA blood velocity values (Fig. 2B). The same is true for gd10, day at which no differences were recorded among the groups for implantation areas (Fig. 1A,B) or UA blood velocity parameters (Fig. 2C). Accordingly, we registered comparable numbers of implantations, abortions and abortion rates in all groups when sacrificed at gd10 (Fig. S2A,B). Thus, BPA applied from day 1 to day 7 of pregnancy does not seem to influence fetal growth until mid-pregnancy. Interestingly, the analysis of implantation size at later stages revealed a negative effect of BPA. Implantation size in BPA-treated mothers was significantly reduced (*P* < 0.001) compared to control mice at gd12 (Fig. 1A,B). UA velocity parameters were unaffected by BPA-treatment at this day (Fig. 2D). At gd14, we did an interesting observation: While most implantations of control mice were too large to fit on the screen of the Vevo 2100 system and could not be measured, implantations of BPA-treated mice were smaller and could be measured and quantified (Fig. S3A,B). UA velocity parameters (Fig. 2E) were unaffected by BPA-treatment at gd14. Placental parameters, placental area (Fig. 3A), thickness (Fig. 3B) and diameter (Fig. 3C) were not affected by BPA-treatment as measured at gd10 and gd14 via ultrasound. No changes in the abortion rate as analyzed at gd14 were registered upon BPA exposure (Fig. S4A,B).

### Fetuses and placentas from BPA-treated mothers were lighter than the controls at day 14 of pregnancy confirming that exposure to BPA during early pregnancy impairs fetal growth

**Figure 4:**
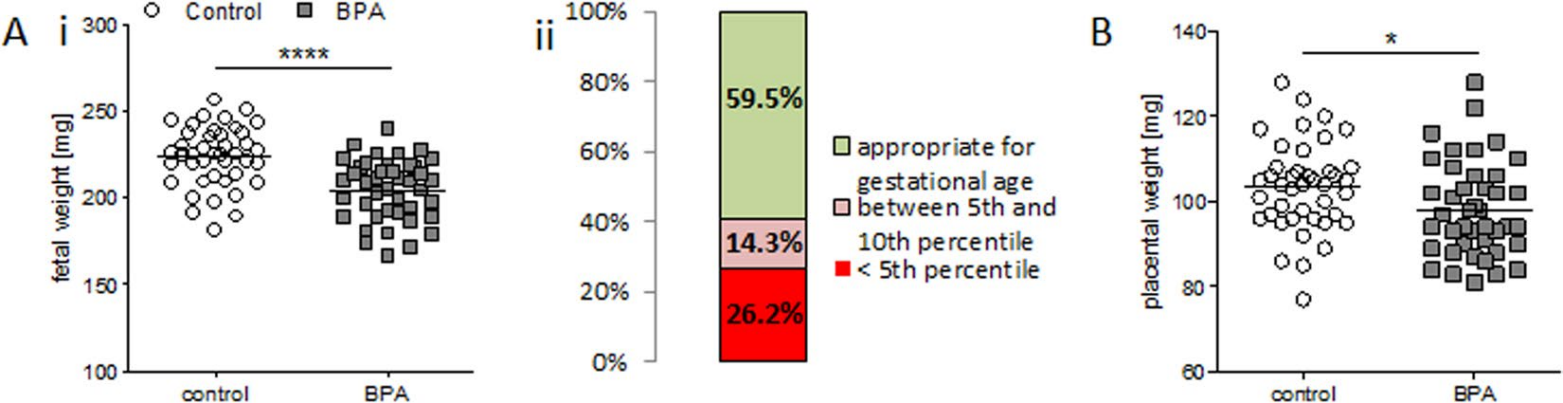
Lower placental weight and intrauterine growth restriction in BPA-treated mice at gd14. Fetal weights (**A**) and placental weights (**B**) from the progenies of C57BL/6J (*n* = 5) or BPA-treated C57BL/6J (*n* = 5) mice at gd14. Fetal parameters are presented as individual values and means. Statistical differences were determined using unpaired t-test (*P < 0.05, ****P < 0.0001). (**A**i) Percentage of fetuses from C57BL/6J and BPA-treated C57BL/6J mice from (**A**ii) whose weight was below the 5^th^ percentile (red); between the 5^th^ and the 10^th^ percentile (pink); or above the 10^th^ percentile (green). gd, gestation day.

Next, the weight of fetuses and placentas was measured after sacrificing pregnant mothers at gd14. Fetal weight from BPA-treated females was significantly reduced (*P* < 0.0001) compared to the control group (Fig. 4Ai). We further organized fetal weight results in percentiles to evaluate the extent of growth retardation. Our results show that 40.5% of fetuses in the BPA group was affected by IUGR as their weight was under the 10^th^ percentile. A percentage of 26.2 of the fetuses was even under the 5^th^ percentile (Fig. 4Aii). The placental weights of conceptuses from BPA-treated mothers were statistically significant reduced (*P* < 0.05) compared to controls (Fig. 4B). Our data show impressively that BPA exposure during early pregnancy, even if short-term, has a dramatic effect on fetal and placental growth, with 40.5% of the fetuses being affected by IUGR.

### Impaired uSA remodeling in BPA-treated animals

**Figure 5:**
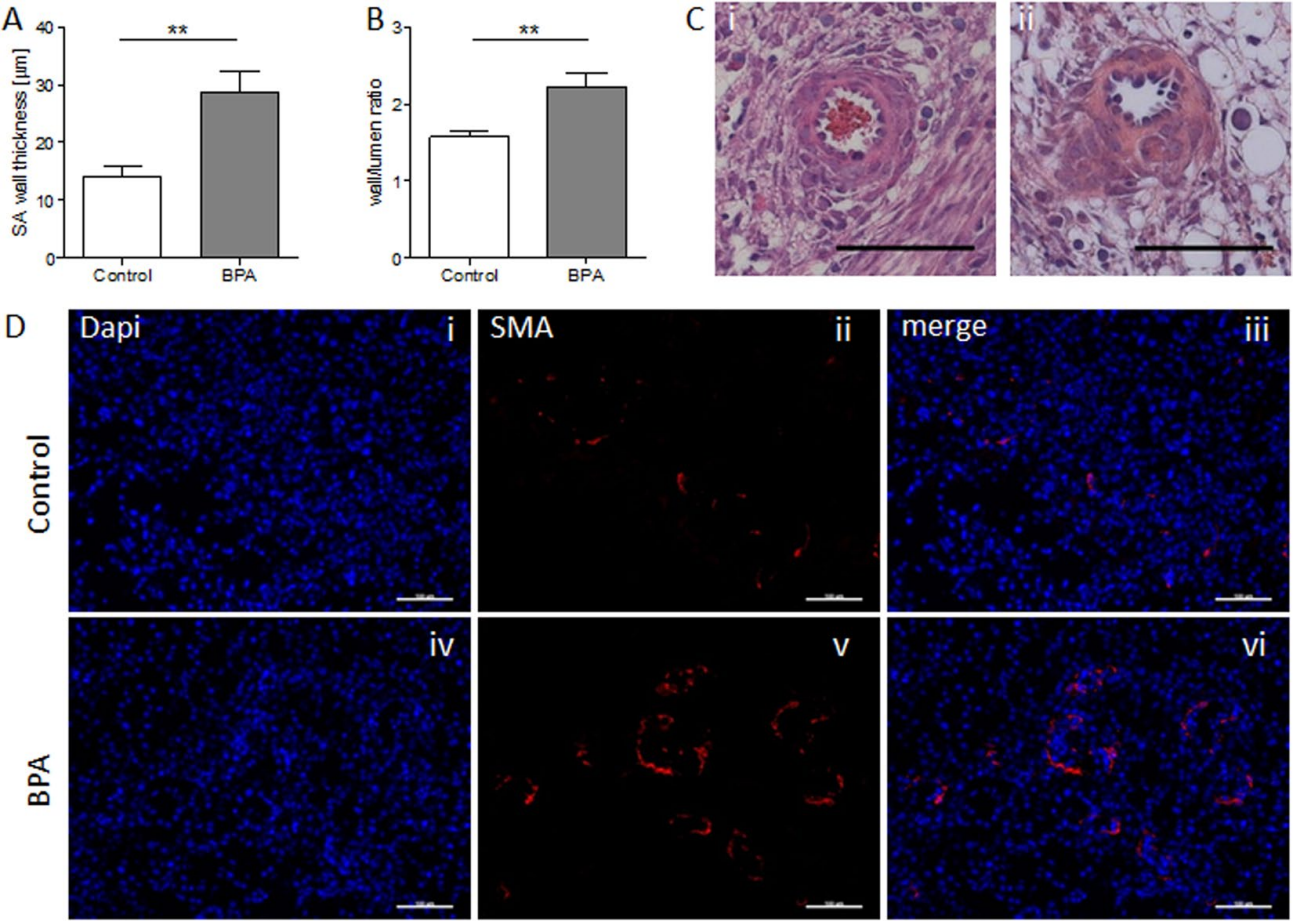
Impaired uSA remodeling of BPA-treated mice at gd10. uSA thickness (**A**) and wall-to-lumen ratios (**B**) from C57BL/6J control (*n* = 7) and BPA-treated C57BL/6J mice (*n* = 5) were calculated by measuring wall and lumen diameters of 3–8 uSAs per mouse. Results are presented as means ± S.E.M. and analyzed by using Mann-Whitney-*U*-test (**P < 0.01). (**C**) Representative images of uSAs from H/E-stained implantations of C57BL/6J (i) and BPA-treated C57BL/6J (ii) are shown (scale bars = 100 µm). (**D**) Representative smooth muscle actin immunfluorescence staining images of SAs from implantations of BL/6J (i-iii) and BPA-treated BL/6J (iv-vi) are shown (scale bars = 100 µm). uSA, uterine spiral artery; gd, gestation day.

**Figure 6:**
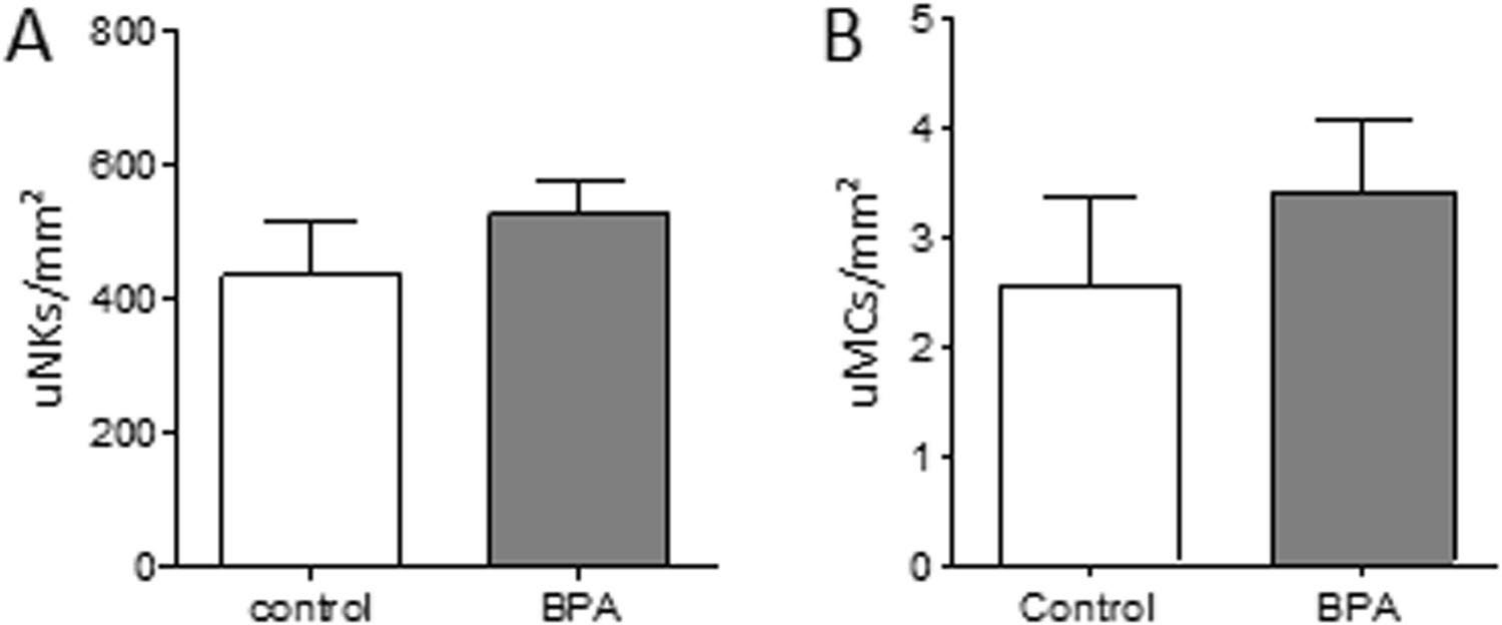
Comparable number of uterine natural killer cells (uNKs) and uterine mast cells (uMCs) of control and BPA-treated mice. (**A**) Number of uNKs per 1 mm^2^ quantified in *Dolichos biflorus agglutinin* (DBA) lectin-stained cross sections of implantations of BL/6J (n = 5) and BPA-treated BL/6J mice (n = 5) at gd10. (**B**) Number of uMCs per 1 mm^2^ quantified in toluidine blue-stained cross sections of uteri of BL/6J (n = 5) and BPA-treated BL/6J mice (n = 4) at gd5. Results are presented as means with SEM. Statistical differences were obtained using Mann-Whitney-*U*- test. uNKs, uterine natural killer cells; gd, gestation day; DBA, *Dolichos biflorus agglutinin;* uMCs, uterine mast cells.

uSA remodeling is a milestone in pregnancy, as it conditions the blood flow from the mother to the fetus. During this process SAs transform from thick-wall arteries into arteries with thin walls and wide lumens. The transformation of the artery wall owes to the loss of uterine smooth muscle cells (SM) that is fundamental to prevent SA vasoconstriction that could interrupt the blood stream to the placenta. IUGR is thought to be a consequence of impaired uSA remodeling^41^. Here, we registered uSA parameters in H/E-stained slides obtained from uterine tissue of BPA-treated and control mice. Our measurements revealed a statistically significant increased (*P* < 0.01) wall thickness (Fig. 5A) and a statistically significant increased (*P* < 0.01) wall-to-lumen ratio (Fig. 5B) in uSA of BPA-treated mothers (Fig. 5Cii) in contrast to controls (Fig. 5Ci). The staining for SM actin (SMA) (Fig. 5D) confirms that more SMA remains visible in SAs of BPA-treated mothers (iv-vi) in contrast to controls (i-iii). The data suggests that BPA has a negative effect on uSA remodeling, a major milestone for fetal development. As it has been recently shown that uNKs and uMCs work together to guarantee an efficient uSA remodeling^39^, we next proceeded to quantify their numbers at the feto-maternal interface. However, neither uNKs nor uMCs (Fig. 6) were affected at least in number by BPA-treatment. Thus, they do not seem to be the target of BPA for the impaired uSA remodeling despite having receptors for estrogens and being able to migrate to the feto-maternal interface in response to hormones^42^.

## Discussion

Well-balanced hormone levels are mandatory for a successful pregnancy. Along with the pregnancy hormone, the human chorionic gonadotropin, estrogen and progesterone are the main actors during gestation. These hormones fulfill their function via binding to progesterone receptors (PR) and estrogen receptors (ER) α, ERβ and ERRγ^43,44^.

The xenoestrogen BPA is classified as an endocrine disruptor as it is able to disturb hormone pathways by binding to their receptors. Although several adverse effects of BPA on reproductive processes have been documented in epidemiologic studies and wildlife, the extent of its effect on pregnancy and the underlying mechanisms in animal models and consequences to human health remain controversial in particular because the results of different studies are contradictory. In particular, little is known about the effects of BPA exposure during early pregnancy and the putative consequences for the growing fetus. In the present study we aimed to dissect the direct consequences of exposure to the contaminant BPA whose main way of contact is via food and beverages^9,10^ immediately after conception and during the early pregnancy stages. On account of this, our experimental approach consisted of exposing pregnant females to BPA during the mentioned early period of pregnancy. The U.S. Environmental Protection Agency (EPA) has published a reference dose (RfD, 0.05 mg/kg/day) for BPA as the acceptable daily intake (ADI) for BPA through food additive use. Oral gavage was selected as the administration route, as in humans the uptake of BPA takes place mainly from food products^6–10^. BPA application took place from day 1 to 7 of pregnancy, being day 0 defined as the day at which a vaginal plug was registered. We analyzed the impact of this allegedly harmless dose of BPA on pregnancy outcome and concentrated on the following read out parameters: implantation outcome, implantation size throughout pregnancy, fetal and placental size as well as fetal and placental weight. We observed that oral exposure to BPA during early pregnancy resulted in growth restricted fetuses. IUGR was further accompanied by impaired remodeling of uterine SAs, evidenced by a higher SA wall thickness in uterus of pregnant mothers that were exposed to BPA compared to the controls.

In our experimental setting, the exposure to BPA during early pregnancy had no effect on implantation number or size as analyzed at day 5. In other words, implantation was successful in mothers previously exposed to BPA. In the literature, negative effects were described in mice that were repeatedly treated with 100 mg/kg BPA/day, a dose that hindered implantation, while 40 mg/kg BPA/day resulted in delayed embryo implantation in mice^45^. A dose of 20 mg/kg BPA/day significantly reduced the number of implantations in rats^46^. These doses are, however, much higher than the reported real exposition doses. Here, and by using the officially maximal allowed BPA dose, we did not observe any effect in the implantation numbers or implantation sizes as analyzed at day 5. We conclude that BPA in the concentration employed here has no major adverse effect on the implantation process itself. We cannot discard, however, that implantation is delayed or the molecular mechanisms dictating the implantation process are negatively modified in a way that their effects become first visible when the fetus is growing bigger. Testing the direct effect of BPA on *in vitro* implantation models should help clarifying this aspect.

After observing no effects of BPA regarding the number of implantations and size of implantations at gd5, we next sought to understand whether the implantations developed normally throughout pregnancy. High-frequency ultrasound is a valuable tool for monitoring fetal growth. Here, we performed ultrasound examinations at gd5, 8, 10, 12, and 14. Measurements of the implantation areas at gd5, 8, and 10 revealed no differences in implantation size between BPA-treated and control animals. Hence, we speculate that BPA did not negatively influence implantation and fetal growth at early to mid-pregnancy.

To understand whether BPA exposure may affect fetal growth at mid and late pregnancy, we analyzed the data obtained for fetal and placenta development at later pregnancy stages. At gd12, implantation sizes in BPA-treated mothers were significantly smaller than the controls. This difference in size between BPA-treated and control animals became particularly obvious at gd14 when implantations from most control mice were too large to fit entirely on the ultrasound screen (just two of them were measureable), whereas ten implantations from BPA-treated mothers could be displayed on the screen in full size and measured. Hence, it appears that BPA exposure affects placental or fetal growth at gd12 in the mouse, which corresponds to mid-gestation in humans. A reduced implantation size can result from either a small placental or fetal size, or both. Although we could not measure statistically significant differences regarding the placental area, -thickness and -diameter at gd10 and gd14 by ultrasound, the analysis at gd14 showed that BPA-exposed mothers have a significant reduced placental weight in contrast to controls. At this point, we do not know the reason as to why placentas from BPA-treated mothers are lighter than those of the controls despite showing similar histologic parameters. One possible explanation is that placentas from the BPA group received less maternal blood than the controls and as blood is soaking the whole placenta, differences in the blood volume reaching it may provoke differences in the weight of the organ. This is however just a speculation as its confirmation is technically impaired. Exposure to BPA affected not only the placental weight but also fetal weight. The progeny of BPA-treated mothers presented IUGR that was manifest at gd14, namely two days after observing smaller implantation areas by high frequency ultrasound. IUGR was evidenced in 40.5% of all fetuses in the BPA group; they showed a weight under the 10^th^ percentile in contrast to pups of the control group at the same gestational age. This fact is even more alarming when considering that 26.2% of all fetuses presented fetal weights that were even below the 5^th^ percentile. We believe that our working model is of high relevance for studying the mechanisms underlying BPA-driven IUGR. This is of particular importance when keeping in mind the dramatic effects resulting from IUGR in humans^47–53^. Snijder *et al*. documented a link between prenatal BPA exposure in women and lower growth rates of their babies^54^. Susiarjo and colleagues recently provided evidences that maternal BPA exposure disrupted imprinted gene expression in the placenta and this was associated with abnormal placental development.

Failure of the placenta to develop normally and reach the expected weight gain can result in maternal chronic hypertension and preeclampsia^55^ but also in fetal disorders^56^. Despite a clear IUGR phenotype in the BPA-treated group in comparison with the animals exposed to vehicle, no differences were detected in UA values between our groups at different pregnancy time points. Based on these observations we suggest that IUGR in this model is not associated with remarkable changes of the Doppler parameters during pregnancy. We cannot, however, discard that the observation period is not long enough or that we may need larger group sizes to detect relevant differences among the groups.

The impact of BPA in fetal growth prompted the question about the mechanisms leading to this phenotype. We observed that the uSAs of BPA-treated mothers had thicker walls than those of the controls. This suboptimal remodeling where smooth muscle cells are still present at multilayer has negative consequences for the blood flow as not only the velocity of blood to be transported is affected and by consequence the amount of blood reaching the placenta, but also the vasoconstrictive ability of the SAs. Two cell types are relevant for uSA remodeling, namely immune cells and trophoblasts. Both are possible targets of BPA as they express hormone receptors and are sensitive to hormones as many *in vitro* studies confirmed^42,57–60^. Trophoblasts need to migrate and replace endothelial cells from the uSAs and immune cells secrete factors that promote trophoblast migration as well as apoptosis of uterine smooth muscle cells^61^. Here, ongoing experiments suggest no major differences in the frequencies of different subsets of innate and adaptive immune cells, neither systemically nor locally. In particular the number of uMCs and uNKs was comparable between both groups. A further relevant factor in the process of SA remodeling is the SM phenotype. The shift from a contractile phenotype to a synthetic one increases the ability of SM to migrate, a process that promotes SA remodeling^62–64^. Substances like platelet-derived growth factor, transforming growth factor-β, Angiotensin II, and nitric oxide can influence the expression of SM phenotype markers^62^. Whether BPA is able to influence the functionality of immune cells so that secrete SM-modulatory mediators or to mediate the degradation of specific ECM molecules, restricting the efficient remodeling of SAs is not known yet and of interest for future studies. Complex study designs are needed to address this question and we hope that our study encourages researchers to do so.

In summary, we present strong evidences that short-time exposure to BPA during early murine pregnancy is sufficient to provoke IUGR in the fetuses; being IUGR preceded by insufficient remodeling of uterine spiral arteries. We propose that BPA can negatively affect fetal development by directly affecting SA remodeling. This alarming fact should prompt further studies on the underlying molecular mechanisms with the final aim of designing strategies tending to prevent such drastic consequences for the unborn.

## Electronic supplementary material

Supplementary Figures S1-S4
