# Supplementary material for: Bisphenol A exposure during early pregnancy impairs uterine spiral artery remodeling and provokes intrauterine growth restriction in mice
Source: Sci Rep. 2018 Jun 15;8:9196. doi: 10.1038/s41598-018-27575-y (PMC6003928; doi:10.1038/s41598-018-27575-y)
Supplement: Supplementary file 1 — Supplementary Figures S1-S4 [file 41598_2018_27575_MOESM1_ESM.doc]

**SUPPLEMENTARY INFORMATION**

**Bisphenol A exposure during early pregnancy impairs uterine spiral artery remodeling and provokes intrauterine growth restriction in mice**

**Authors:** Judith Elisabeth Müller^1#^, Nicole Meyer^1#^, Clarisa Guillermina Santamaria^2^, Anne Schumacher^1^, Enrique Hugo Luque^2^, Maria Laura Zenclussen^1,2^, Horacio Adolfo Rodriguez^2^*, Ana Claudia Zenclussen^1^*

# shared first authorship, * shared last authorship

**Affiliations:**

^1^ Experimental Obstetrics and Gynecology, Medical Faculty, Otto-von-Guericke University, Magdeburg, Germany

^2^ Universidad Nacional del Litoral, Facultad de Bioquímica y Cs. Biológicas, Instituto de Salud y Am biente del Litoral, UNL-CONICET, Santa Fe, Argentina

~ Corresponding author: Ana C. Zenclussen, Experimental Obstetrics and Gynecology, Medical Faculty, Otto-von-Guericke-University, Gerhart-Hauptmann Str. 35, 39108 Magdeburg, Germany. Phone: +49391/6717460, Fax: +49391/6717440, E-mail: [ana.zenclussen@med.ovgu.de](mailto:ana.zenclussen@med.ovgu.de)

**Short title:** BPA provokes IUGR

**Keywords**: Bisphenol A, pregnancy, intrauterine growth restriction, spiral arteries


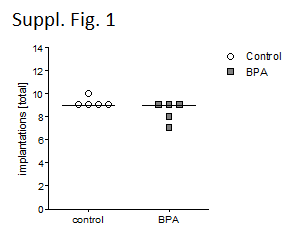


**Suppl Fig. 1: Comparable number of implantations of control and BPA-treated mice at gestation day (gd) 5.** Number of implantations from progeny of C57BL/6J mice (n=5) and BPA-treated C57BL/6J mice (n=5) at gd5. Results are presented as individual values and medians. Statistical differences were obtained using Mann-Whitney-*U* test. gd, gestation day.


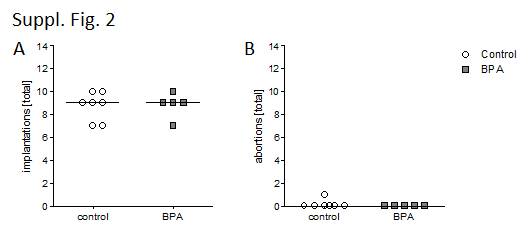


**Suppl Fig. 2: Comparable number of implantations and abortions of control and BPA-treated mice at gestation day (gd) 10.** Number of implantations **(A)** and abortions **(B)** from progeny of C57BL/6J mice (n=7) and BPA-treated C57BL/6J mice (n=5) at gd10. Results are presented as individual values and medians. Statistical differences were obtained using Mann-Whitney-*U* test. gd, gestation day.


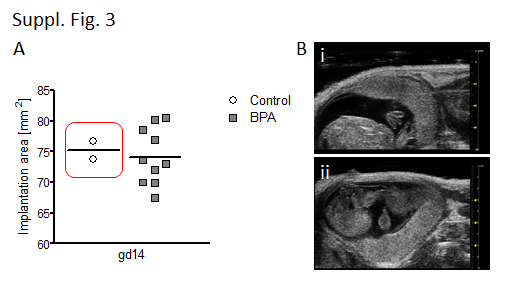


**Suppl Fig. 3: Implantation sizes in control and BPA-treated mice at gestation day (gd) 14. (A)** Implantation areas of single implantations of control (mice: n=5; measureable implantations: n=2) and BPA-treated BL/6J (mice: n=5; measureable implantations: n=10) mice at gd14. Implantation sites are presented as individual values and means. Determination of statistical difference was not possible due to the amount of just 2 measureable parameters in the control group out of 24. **(B)** Representative 2D greyscale ultrasound images from control (i) and BPA-treated BL/6J mice (ii). gd, gestation day.


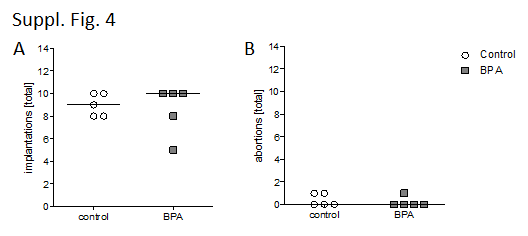


**Suppl Fig. 4: Comparable number of implantations and abortions of control and BPA-treated mice at gestation day (gd) 14.** Number of implantations **(A)** and abortions **(B)** from progeny of C57BL/6J mice (n=5) and BPA-treated C57BL/6J mice (n=5) at gd14. Results are presented as individual values and medians. Statistical differences were obtained using Mann-Whitney-*U* test. gd, gestation day.
